# Are Adult Crambid Snout Moths (Crambinae) and Larval Stages of Lepidoptera Suitable Tools for an Environmental Monitoring of Transgenic Crops? — Implications of a Field Test

**DOI:** 10.3390/insects2030400

**Published:** 2011-08-10

**Authors:** Andreas Lang, Matthias Dolek, Bernhard Theißen, Andreas Zapp

**Affiliations:** 1 Institute of Environmental Geosciences, University of Basel, Bernoullistrasse 30, Basel CH-4056, Switzerland; 2 Büro Geyer & Dolek, Obere Dorfstr. 16, Wörthsee D-82237, Germany; E-Mails: Matthias.Dolek@Geyer-und-Dolek.de (M.D.); a.zapp@naturplan.net (A.Z.); 3 gaiac – Research Institute for Ecosystem Analysis and Assessment e.V., RWTH Aachen University, c/o Institute of Environmental Research - Biology V, Worringerweg 1, Aachen D-52056, Germany; E-Mail: theissen@gaiac.rwth-aachen.de

**Keywords:** monitoring methods, transect count, visual search, beating sample, standardization, cost-efficiency, GMO, non-target organism

## Abstract

Butterflies and moths (Lepidoptera) have been suggested for the environmental monitoring of genetically modified (GM) crops due to their suitability as ecological indicators, and because of the possible adverse impact of the cultivation of current transgenic crops. The German Association of Engineers (VDI) has developed guidelines for the standardized monitoring of Lepidoptera describing the use of light traps for adult moths, transect counts for adult butterflies, and visual search for larvae. The guidelines suggest recording adults of Crambid Snout Moths during transect counts in addition to butterflies, and present detailed protocols for the visual search of larvae. In a field survey in three regions of Germany, we tested the practicability and effort-benefit ratio of the latter two VDI approaches. Crambid Snout Moths turned out to be suitable and practical indicators, which can easily be recorded during transect counts. They were present in 57% of the studied field margins, contributing a substantial part to the overall Lepidoptera count, thus providing valuable additional information to the monitoring results. Visual search of larvae generated results in an adequate effort-benefit ratio when searching for lepidopteran larvae of common species feeding on nettles. Visual search for larvae living on host plants other than nettles was time-consuming and yielded much lower numbers of recorded larvae. Beating samples of bushes and trees yielded a higher number of species and individuals. This method is especially appropriate when hedgerows are sampled, and was judged to perform intermediate concerning the relationship between invested sampling effort and obtained results for lepidopteran larvae. In conclusion, transect counts of adult Crambid Moths and recording of lepidopteran larvae feeding on nettles are feasible additional modules for an environmental monitoring of GM crops. Monitoring larvae living on host plants other than nettles and beating samples of bushes and trees can be used as a supplementary tool if necessary or desired.

## 1. Introduction

National and international regulations govern the risk assessment of genetically modified organisms (GMO) before release and the necessity for a post-release monitoring plan (e.g., [1-3]). In the European Community, the EU Directive 2001/18/EC stipulates the implementation of a monitoring plan to monitor any adverse effects of GMO on human health or the environment [4], while the Council Decision 2002/811/EC provides further specifications about the objectives, general principles and framework for such monitoring plans [5].

Butterflies and moths (Lepidoptera) have frequently been suggested for such a GMO monitoring [6,7], because transgenic crops can have adverse effects on this insect group, both directly and indirectly. For example, pollen of Bt (*Bacillus thuringiensis*) maize toxic to pest Lepidoptera can drift by wind onto host plants of non-target lepidopteran larvae, and the non-target larvae may be adversely affected by consuming this pollen [8,9]. The application of broad spectrum herbicides, such as glyphosate or glufosinate-ammonium, in combination with genetically modified herbicide-tolerant crops is likely to change the herbicide regime, which can reduce the weed community within fields and in field margins, in turn affecting larval and adult butterflies associated with those food plants [10,11]. Lepidoptera appear to be suitable for GMO monitoring purposes, because they are considered to be good field indicators and are frequently used in environmental monitoring schemes [12]. In addition, there exists a standardized methodology to monitor adult Lepidoptera, e.g., the transect count method for butterflies and light traps for night-active moths. Both methods are widely accepted and applied [13,14].

The German Association for Engineers (VDI = Verein Deutscher Ingenieure) developed and published scientific guidelines specifically for the monitoring of GMO effects on Lepidoptera [7]. These guidelines describe standardized recording methods for the monitoring of adult butterflies and moths, e.g., the transect count method and the use of light traps adapted to the utilization in agricultural landscapes. In addition, two methods less frequently applied were included, (i) the recording of adult Crambid Snout Moths (Pyraloidea, Crambidae: Crambinae) during transect counts; and (ii) the recording of larvae of certain Macrolepidoptera species. The monitoring of stationary larvae of Lepidoptera has two advantages in comparison to the recording of the mobile adults: the larval appearance provides better evidence for an indigenous occurrence of the species, and potential GMO effects could be better attributed to the specific site. Further, the stage potentially affected by lepidopteran-specific Bt maize would be the larval stage and not the adult one [15]. The Crambid Snout Moths (Crambinae) are an important subfamily of the Pyralidae of about 80 species occurring in Central Europe [16], and some species are often abundant in grassy habitats. The adults are relatively small with a wing-span of about 14–27 mm and the labial palps project forward forming a “snout”. The adults frequently take a typical posture sitting head-down with folded wings on grasses. The larvae usually feed on roots and stems, commonly on grasses [16,17]. According to the VDI approach, including the monitoring of adult Crambid Snout Moths could improve the recorded data set and monitoring results, in particular because butterfly richness can be low on grassy field margins in intensively managed agro-ecosystems [18,19].

However, the recording of adult Crambid Snout Moths and of Lepidoptera larvae has not previously been standardized or tested for GMO-monitoring purposes. Here, we report on a test of the two methods in three regions of Germany, following the methodological description of VDI [7]. The main goal of the study was to assess the feasibility of these approaches in principle (and not to carry out a complete monitoring program). Therefore, we conducted representative field surveys in order to evaluate the practicability and the effort-benefit ratios. The following questions were of particular interest: (i) is it possible and practicable to record Crambid Snout Moths in combination with transect counts of adult butterflies; (ii) how laborious is an additional counting of Crambinae; (iii) what results can be obtained with a monitoring of lepidopteran larvae; and (iv) is a larval monitoring practicable, *i.e.*, what is the relationship between effort and recorded results?

## 2. Experimental Section

### 2.1. Study Sites

In 2008, field surveys were carried out in three different regions in Germany: South Upper Rhine (Südlicher Oberrhein), Upper Franconia (Oberfranken) and Jülich Boerde (Jülicher Börde). The three sampling locations of the Upper Rhine were near the villages Rheinweiler, Blansingen and Efringen-Kirchen (about 10–20 km north of Basel, Switzerland), the two locations of Upper Franconia were Bindlacher Berg and Benk (north of Bayreuth), and the two locations in the Jülich Boerde were near Koslar and Stetternich (between Aachen and Cologne). All sampling locations were within arable land and were situated in field margins along maize fields in the regions South Upper Rhine and Upper Franconia, and along winter wheat and sugar beet fields in the Jülich Boerde.

### 2.2. Monitoring of Crambid Snout Moths

Along crop fields, transect routes were established, walked in regular intervals and all observed adult butterfly and Crambid snout moth specimens recorded, *i.e.*, species of Papilionoidea, Hesperioidea as well as Crambinae. The monitoring approach followed a strictly standardized design according to the VDI guidelines: defined favorable weather conditions were a precondition, monitoring was to be conducted between 10 am and 5 pm, walking pace and spatial observation radius was fixed, transects were divided into 50m sections and additional parameters were recorded such as land use and flowering aspect. For the transect counts, the field recording sheets given by the VDI guidelines were used (for more details see [7]). The transect counts were carried out between 9 June and 9 September 2008. Transect lengths as well as recording dates and intervals differed between the study regions (Table 1).

### 2.3. Monitoring of Lepidopteran Larvae

The larvae of Macrolepidoptera were recorded by two different methods: visual searching and beating samples.

A directed visual search for recording lepidopteran larvae has been described by Hermann [20]. In the VDI guidelines this method was adapted for standardized monitoring purposes [7]. The general approach is a plant-oriented search, *i.e.*, the search is directed to the known larval host plants. A crucial point of the directed search is to check only suitable host plants, *i.e.*, suitable for the larvae due to the microclimatic condition as well as nutritional situation and growth state of the plant. The VDI standardization included a defined number of plants to be searched, defined searching times per host plant (populations), and adequate weather conditions. For the visual searches, the field recording sheets given by the VDI guidelines were used (for more details see [7]). Visual searches were carried out between 9th June and 9th September, and differed among regions due to differing climatic conditions and species occurrence (Table 2).

Beating samples is a widely applied approach to collect arthropods from bushes and trees [14]. The standardized use for sampling lepidopteran larvae occurring on bushes and trees is described in the VDI guidelines [7]. The general approach is to beat branches with a stick and to collect and count the specimens caught on a collecting sheet placed below. The VDI standardization of the method included favorable and dry weather conditions during sampling events, beating technique (two hits per branch), and sampling of 100 branches (if possible) per location (e.g., a hedgerow, or a row of trees). For the beating samples the field recording sheets given by the VDI guidelines were used (for more details see [7]). Beating samples were carried out between 9th June and 11th August, and differed among regions due to differing climatic conditions and species occurrences (Table 2).

## 3. Results

### 3.1. Monitoring of Crambid Snout Moths

**Figure 1:**
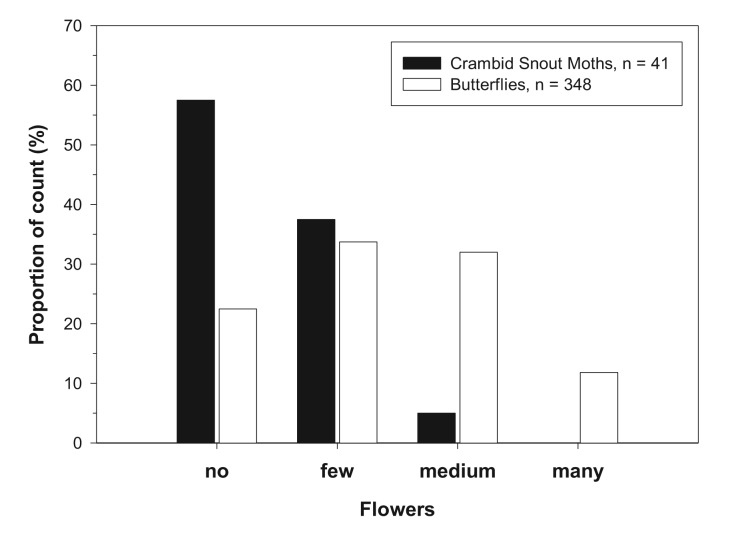
Relationship between flowering aspect and occurrence of butterflies and Crambid Snout Moths in the region South Upper-Rhine. Given is the respective proportion (%) of each count in relation to the number of flowers present in the field margins.

Overall, 30 lepidopteran species were recorded by the transect counts: 25 butterfly species and five species of Crambinae (Table 3, Appendix 1). Most individuals and species were recorded in the South Upper-Rhine, reflecting the higher number of sites, the longer transects and the general higher biodiversity in this region. Crambid Snout Moths were recorded in four out of the seven sampling locations, *i.e.*, in 57% of the field margins. On average, 1.6 species and 16.7 individuals of Crambid Snout Moths were recorded per field margin, the proportion of the total Lepidoptera count being 10% for species number and 16% for individual abundance. Especially in the species-poorer and intensively managed regions Upper Franconia and Jülich Boerde, the fraction of Crambid Snouth Moths was considerably high (Table 3). The most abundant species of the Crambinae was *Crambus perlella* (Table 4), occurring in all study regions and making up a considerable part of the overall Lepidoptera count (compare Table 4 and Appendix 1). In the Upper Rhine, occurrence of Lepidoptera was analyzed in relation to the flowering aspects, *i.e.*, in relation to the number of flowering plants present in the respective field margins. In general, butterflies were more abundant when flowers were present in field margins (Figure 1). Crambid Snout Moths showed no positive association with the number of flowering plants, in contrast, they were more abundant on field margins without flowers (Figure 1).

### 3.2. Monitoring of Lepidopteran Larvae

Overall, visual searching of Lepidoptera larvae detected the larvae of nine species. On *Urtica dioica* host plants, three species were detected: *Inachis io*, *Vanessa atalanta* and *Pleurotypa ruralis* (Table 5). The larvae of *I. io* and *P. ruralis* were found in all three regions. The number of larvae of *I. io* in communal nests ranged from 40–160 larvae per web. The searching times for webs of *I. io* were 18.6 minutes/web (South Upper-Rhine), 8.8 minutes/web (Upper Franconia) and 26.4 minutes/web (Jülich Boerde).

Of plants other than *U. dioica*, 17 potential host plants were searched (Table 5), which yielded 27 larvae of six species: *Pieris rapae* (Pieridae), *Emmelia trabealis* (Noctuidae), *Tyta luctuosa* (Noctuidae), *Tyra jacobaeae* (Arctiidae), *Phragmatobia fuliginosa* (Arctiidae) and an unidentified moth larva on *Sedum telephium*. The searching times per larva were 73.5 minutes/larva (South Upper-Rhine), 109.4 minutes/larva (Upper Franconia) and 3.25 minutes/larva (Jülich Boerde). The low searching time in the Jülich Boerde was caused by a single recording event of 20 aggregated larvae of *T. jacobaeae* at one occasion.

During searching of larvae, eggs of Lepidoptera were also detected: 18 eggs of *Cyaniris semiargus* (Lycaenidae) on *Trifolium pratense* in the South Upper-Rhine, and 128 eggs of *Pieris rapae*/*brassicae* (Pieridae) on *Brassica napus* in Upper Franconia.

In addition, it was noted that larvae of the Ermine Moths (Yponomeutidae) were occurring occasionally, feeding on blackthorn bushes (*Prunus spinosa*). Most larvae had already pupated due to the late sampling date, but their communal nests could still be counted. In the South Upper-Rhine, 91 yponomeutid webs were recorded by searching an area of 67m^2^ densely covered with blackthorns (searching time of 15 minutes), while in Upper Franconia 17 yponomeutid webs were counted by checking 50 single blackthorn bushes (searching time of 30 minutes).

Overall, the beating samples yielded 30 larvae (Table 6) which belonged to nine species: *Eilema* sp. (Arctiidae), *Notodonta ziczac* (Notodontidae), *Clostera pigra* (Notodontidae), *Craniophora ligustri* cf. (Noctuidae), *Catocala* sp. (Noctuidae), *Lomaspilis marginata* (Geometridae), *Chloryclysta siterata* (Geometridae), *Cabera exanthemata* (Geometridae) and *Yponomeuta* sp. (Yponomeutidae). Of the overall catch, 33% of the individuals could be attributed to the species or genus, respectively, while 66% of the individuals could not be identified. Handling time of one double-hit was roughly one minute including sorting and collecting the catch (but excluding species identification), and collection time per larva was on average 20 minutes.

## 4. Discussion

### 4.1. Monitoring of Crambid Snout Moths

Crambid Snout Moths *in partim* proved to be a suitable extension to the transect counting of butterflies, requiring not much additional effort. When occurring, they were abundant and made up a substantial part of the individuals. Also, they were widespread, occurring in all three regions studied, in particular *C. perlella*. By including Crambid Snout Moths in GMO monitoring, the data set would be increased, thus improving the power of subsequent statistical analyses. This may especially hold true for very short transects along small fields, where recorded Lepidoptera abundance in total will be lower [21]. Crambid Snout Moths are not very mobile and effects on them could be more attributed to the site. Adults do not feed on flowers, and the larvae feed on grass [16,17], hence the moths can be still abundant in grassy habitats or agricultural field margins without flowers (this study), where butterfly numbers typically decrease [11]. However, Crambid Snout Moths were not detected in each field margin and their species richness was relatively low. Although this might be due to the limited scope of the study, it may also indicate that Crambid Snout Moths are only suitable as a supplementary monitoring module, but not as a stand-alone tool.

There are some minor handicaps involved with the monitoring of Crambid Snout Moths. The moths must be captured with a net for species identification. But in contrast to the Skippers (Hesperiidae), which must be netted too, catching Crambid Snout Moths is much easier. In the field, the moths themselves can easily be spotted and identified as Crambid Snout Moths by their typical head downwards posture in the vegetation (Kolbeck, pers. comm., personal observation). There exists no field identification guide for the Crambinae. Identification must be done by consulting several books, pictures from the internet, or newsgroups of experts. However, species number in arable land is low, and experienced butterfly experts should be able to cope with the respective species set of Crambinae after some practice. Adult Crambid Moths will not necessarily indicate a reduction of flowering plants like butterflies, and larvae may possibly not indicate aerial pollutants as they can live quite hidden in the vegetation, e.g., in stems of grasses [17].

### 4.2. Monitoring of Lepidopteran Larvae

It appears possible to include the recording of Lepidoptera larvae for GMO monitoring purposes. However, the results and efficiencies differ depending on the applied methods. Nettles, *Urtica* plants, are quite abundant in arable land and field margins [22], consequently larvae of the Peacock Butterfly (*I. io*) were easy to find and to monitor. However, it has to be noted that Peacock larvae of earlier stages spin communal nests and occur aggregated in batches, which can also influence the distribution of older larvae even after separation of the caterpillars (personal observation). The occurrence of further larvae of common lepidopteran species on nettles adds to the usefulness of monitoring a group of “nettle Lepidoptera”, *i.e.*, *V. atalanta*, *Aglais urticae*, *Araschnia levana*, *Polygonia c-album*, *Hypaena proboscidalis*, and *P. ruralis*. It has to be kept in mind that the different species differ in detectability and required monitoring effort, e.g., while communal nests of *I. io* and *A. urticae* are quite conspicuous, the single larvae of *P. ruralis*, which are hidden in nettle leaf bags, are more difficult to spot. Amazingly, larvae of the Small Tortoiseshell (*A. urticae*) could not be recorded in this study, although it is a common species in agricultural land [22]. However, in 2008, numbers of Small Tortoiseshells were generally low in Germany [23]. Further, a monitoring program more extensive than the present study will yield a much larger data set and more robust results. Very similar to the Peacock Butterfly, the communal nests of larvae of Ermine Moths (Yponomeutidae) were easy to detect in bushes and hedgerows along fields.

Generally, visual search of host plants other than *Urtica* was laborious. Searching time was higher and recorded number of larvae lower in comparison with the other studied approaches (*i.e.*, visual search on nettles and blackthorn, and beating samples). In two regions, searching time for one larva was longer than one hour, while the low searching time in the Jülich Boerde was unusual and due to a single recording event of aggregated larvae. Therefore, visual search of host plants appears less suitable for a general monitoring, and may be used in a more case-specific and supplementary way, e.g., if certain species (groups) are to be monitored for protection purposes or if some species are known to occur abundantly. By all means, species-specific knowledge on larval biology and occurrence is paramount, or must be acquired beforehand.

Beating samples performed intermediate in terms of invested sampling effort in relation to recorded results. However, sampling success of this study suffered from the fact that some common and abundant species could not be recorded due to the later season sampling date (e.g., *Operophtera brunata*, *Orthosia gothica*, *Orthosia incerta*, *Orthosia cerasi*, *Cerastris rubicosa*). The method is convenient to apply, and especially suitable to sample bushes, trees and hedgerows where a visual search is often impractical. Beating samples collected many species of Geometrid Moths (Geometridae), which can sometimes not be identified to a species level. These and other unidentifiable specimens must be reared to adulthood for identification, which causes additional effort, and is sometimes not successful as the larvae may die before reaching adulthood. On the other hand, sampling and identification efficiency will improve if monitoring persons are instructed and gain experience (cf. [7]).

As a general rule, the monitoring of Lepidoptera larvae must be planned and analyzed much more on a regional level. The occurrence of larvae is strongly affected by the available and suitable host plants, habitat fragmentation, regional weather, micro-climatic conditions or management practice, to name just a few relevant factors. These factors are unlikely to be the same over a wider geographic area, and/or are difficult to standardize and to harmonize among study sites.

## 5. Conclusions

Crambid Snout Moths (Crambinae) *in partim* are a useful supplement for the transect counts of adult butterflies. Crambinae are wide-spread, common and abundant in agricultural land, are easy to catch and their species are easily identified. Especially in intensively managed agricultural habitats, the Crambinae might be more prevalent than other Lepidoptera species. In conclusion, we recommend the inclusion of Crambid Snout Moths for the monitoring of potential GMO effects on Lepidoptera.

Visual searching for lepidopteran larvae may also be suitable for a monitoring of possible GMO effects, if the involved host plants and Lepidoptera species are common and abundant in arable land, and if sufficient knowledge on the biology of these larvae exist. As this might not always be the case, we recommend the monitoring of *Urtica* plants and associated larvae of “nettle Lepidoptera”. Searching of host plants other than nettles and beating samples of bushes and trees can be used as a monitoring tool if necessary or desired. In particular, beating samples are recommended if hedgerows are to be studied and a higher number of larvae should be collected.

We acknowledge that the data base of the present study is small, and further research on monitoring of Crambid Snout Moths and lepidopteran larvae is necessary and would be welcomed, including the study of more species, more host plants, more regions and more consecutive study seasons.

## Figures and Tables

**Table 1:** Transect counts in the three study regions in 2008. Figures of “Transect dates” refer to the month of recording, and B = Beginning, M = Mid, E = End.

**Region**	**Site**	**Transect length**	**Transect dates**
South Upper-Rhine	Rheinweiler	1000 m	B6, E6, B7, B8, B9
	Blansingen	1000 m	E6, M7, B8, B9
	Efringen-Kirchen	1000 m	E6, M7, B8, B9
Upper Franconia	Bindlacher Berg	300 m	B6, M6, M7, E7
	Benk	300 m	B6, M6, M7, E7
Jülich Boerde	Koslar	300 m	B6, B7, M8, B9
	Stetternich	250 m	B6, B7, M8, B9

**Table 2:** Monitoring lepidopteran larvae in three study regions in Germany in 2008: visual search and beating samples. Figures of dates refer to the month of recording, and B = Beginning, M = Mid, E = End.

**Region**	**No. of sites**	**Dates of visual search**	**Dates of beating samples**
South Upper-Rhine	3	M6, E6, M8, E8	M6, E6, M8
Upper Franconia	2	B6, M6, E7	B6, M6, M7, E7
Jülich Boerde	2	B6, B7, M8, B9	B6, M6, B7

**Table 3:** Total Lepidoptera count of the transect counts for the three study regions. Note that the regions differ in number of sites, in transect length and in number of visits (see Table 1).

	**South Upper-Rhine**	**Upper Franconia**	**Jülich Boerde**
Butterfly individuals (n)	348	59	47
Snout moth individuals (n)	41	54	22
Butterfly species (n)	22	12	7
Snout moth species (n)	3	4	1

**Table 4:** Species and abundance of Crambid Snout Moths recorded by the transect counts. Note that the regions differ in number of sites, in transect length and in number of visits (see Table 1).

	**South Upper-Rhine**	**Upper Franconia**	**Jülich Boerde**
*Agriphila tristella*	1		
*Agriphila straminella*	5	5	
*Crambus perlella*	34	45	22
*Crambus lathoniellus*		1	
*Chrysoteuchia culmella*	1	3	

**Table 5:** Visual search of Lepidoptera larvae in the three study regions (excluding blackthorn bushes). Note that the regions differ in sampling intensity (see Table 2).

	**South Upper-Rhine**	**Upper Franconia**	**Jülich Boerde**
Urtica *host plants*			
Searched plants (m^2^)	518	79	256
Searching time (min)	149	88	290
Larval nests of *Inachis io* (n)	8	10	11
Larvae of *Vanessa atalanta* (n)	9		
Larvae of *Pleurotypa ruralis* (n)	1	1	11
Species (n)	3	2	2
*Host plants other than* Urtica			
Searched plant species	*Convolvulus arvensis*, *Coronilla varia*, *Galium mollugo*, *Lotus corniculatus*, *Medicago lupulina*, *Medicago sativa*, *Trifolium pratense*, *Trifolium repens*, *Vicia cracca*, *Brassica napus*	*Brassica napus*, *Cirsium* sp., *Galium verum*, *Linaria* sp., *Matricaria* sp., *Sedum telephium*	*Artemisia vulgaris*, *Senecio jacobaeae*
Searched plants (n)	290	1159	85
Searching time (min)	147	547	65
Larvae (n)	2	5	20
Species (n)	1	3	1

**Table 6:** Beating samples of Lepidoptera larvae in the three study regions. Sampling location denotes one branch with one double-hit. Note that the regions differ in sampling intensity (see Table 2).

	**South Upper-Rhine**	**Upper Franconia**	**Jülich Boerde**
Sampled bushes and trees	*Prunus spinosa*, *Salix* spp., *Fraxinus excelsior*	*Prunus spinosa*, *Crataegus monogyna*.	*Crataegus monogyna*, *Salix* spp., *Populus tremula*, *Quercus robur*, *Rosa* sp.
Sampling locations (n)	291	40	155
Larvae (n)	23	4	3
Species (n)	4	2	3

## Appendix

**Appendix 1:** Total count of butterflies (transect count) in the three study regions in 2008. Note that the sites differ in transect lengths and in number of visits (see Table 1).

	**South Upper-Rhine**			**Upper Franconia**		**Jülich Boerde**	

	**Rheinweiler**	**Blansingen**	**Efringen-Kirchen**	**Benk**	**Bindlacher Berg**	**Koslar**	**Stetternich**
*Aphantopus hyperantus*	3		1	2			10
*Carcharodus alceae*		2					
*Coenonympha pamphilus*	1	3	1	19			
*Colias crocea*				6			
*Colias hyale/alfacariensis*		4	12				
*Cupido argiades*	1	2					
*Cyaniris semiargus*		3					
*Cynthia cardui*	1						
*Gonepteryx rhamni*		1					
*Inachis io*	4	1		3	2		
*Issoria lathonia*					1		
*Lasiommata megera*	3	5	4				
*Leptidea sinapis/reali*		1		2			
*Lycaena phlaeas*							1
*Maniola jurtina*	45	51	4	2			11
*Melanargia galathea*	32	6	6	3			
*Ochlodes venata*	2		1				
*Pararge aegeria*	7						
*Pieris napi*		3	5				4
*Pieris rapae*	3	32	42		4		4
*Pieris sp.*		6	10				9
*Polyommatus icarus*	1	10		5			
*Pyronia tithonus*	9	1				1	5
*Thymelicus acteon*	1						
*Thymelicus lineola*	1	14		2			
*Thymelicus sp.*			1				
*Vanessa atalanta*			1			2	
*Unidentified*			1				
